# Exploring Spatial Variability in the Relationship between Long Term Limiting Illness and Area Level Deprivation at the City Level Using Geographically Weighted Regression

**DOI:** 10.3934/publichealth.2015.3.426

**Published:** 2015-07-31

**Authors:** Karyn Morrissey

**Affiliations:** 1 Department of Geography and Planning, University of Liverpool, L69 7ZT, UK

**Keywords:** geographically weighted regression, area level deprivation, long term limiting illness

## Abstract

Ecological influences on health outcomes are associated with the spatial stratification of health. However, the majority of studies that seek to understand these ecological influences utilise aspatial methods. Geographically weighted regression (GWR) is a spatial statistics tool that expands standard regression by allowing for spatial variance in parameters. This study contributes to the urban health literature, by employing GWR to uncover geographic variation in Limiting Long Term Illness (LLTI) and area level effects at the small area level in a relatively small, urban environment. Using GWR it was found that each of the three contextual covariates, area level deprivation scores, the percentage of the population aged 75 years plus and the percentage of residences of white ethnicity for each LSOA exhibited a non-stationary relationship with LLTI across space. Multicollinearity among the predictor variables was found not to be a problem. Within an international policy context, this research indicates that even at the city level, a “one-size fits all” policy strategy is not the most appropriate approach to address health outcomes. City “wide” health polices need to be spatially adaptive, based on the contextual characteristics of each area.

## 1. Introduction

Socioeconomic inequalities in health status are observed in all countries [1]. In Europe, lower socioeconomic position and measures of social and material deprivation are associated with greater morbidity and mortality [2]–[4]. The existence of this negative relationship between the socioeconomic status and the incidence or mortality rates has been demonstrated for many health outcomes [2], including low birth weight [5], cardiovascular diseases and diabetes [6] and respiratory diseases [7]. National level analyses of health are beneficial, as they highlight the main determinants of health for a country and allow for cross-country analysis and comparisons. However, research in health geography, epidemiology and public health, has led to an increasing recognition among practitioners and policymakers that space and health are not mutually exclusive; people and their health are shaped by the places in which they live and inhabit on a regular basis [8],[9]. This is in part because people with similar characteristics, such as income level, age and employment status, cluster together [10] and in part because individuals living in the same neighbourhood are subject to common contextual influences [10]–[12].

With regard to contextual influences, research has found that rates of area level deprivation may lead to disparities in health outcomes by shaping differential access to resources and physical environments that may in turn affect behavioural and psychosocial risk factors for poor health outcomes [9], [12]–[14]. However, Curtis et al., [9] caution that ‘area deprivation’ and ‘health’ are multifaceted terms and may be associated in varying ways. Different aspects of area deprivation may show varying associations with diverse health outcomes. For example, much of the literature on social capital and health inequalities [15] is based on the hypothesis that social relationships such as lack of trust, low levels of social support and restricted social networking, are as important for health outcomes in some instances as material poverty at the area level. Using a variety of spatially disaggregated data and statistical modeling techniques, including ecological regression and multi-level models (MLM) studies have made important contributions to our understanding of how geography and deprivation jointly shape the distribution of different morbidities [9],[15]. These studies have taken great care to encompass a range of individual and area level data associated with health outcomes such as deprivation, indicators of social capital and cohesion and area level infrastructure. However these studies have largely been based on the implied assumption that the relationship between contextual factors and health is spatially homogeneous, i.e. that area based characteristics operate in a similar manner across space. This is a necessary condition for the use of global regression models [14], [16]. Multilevel models, while distinguishing one area from another model space as a discrete entity. Yet, non-stationarity, referring to the variation in relationships across space is a common phenomenon in any geographical based research [17], [18]. Space is continuous and the characteristics of an area will influence the characteristics of its neighbouring areas [17], [19]. Thus, both single level ecological models and nested models, in failing to account for spatially varying relationships across place may lead to biased parameter estimates, misleading conclusions, and ineffective interventions.

Over the last two decades a local-based regression technique, Geographically Weighted Regression (GWR) has been gained popularity for exploring spatial non-stationarity among data [17], [18]. GWR allows parameter estimates to vary locally, unlike global models where these estimates remain constant. Briefly, GWR fits local regression at each location by applying a weighting scheme (based on a kernel function), which gives more weight to neighbouring locations [20]. The results therefore emphasise the spatial patterning of relationships [14], [16]. To date, few studies have employed GWR to examine contextual influences on health outcomees; however there are notable exceptions particularly in the obesity literature [14], [21], [22] and in health outcome research in the USA [13]. However, while each of these studies provided insight in spatial heterogeneity in health outcomes they each used highly aggregated, spatial data. Within these theoretical and modelling contexts, the aim of this research is to extend a more geographically nuanced modeling framework within the health modelling community. Specifically, this study contributes to the urban health literature by investigating whether the impact of area level deprivation, a significant determinant of poor health outcomes in previous research in the UK [23], varies at the small area level in a relatively small, UK city.

## 2. Data

This analysis uses secondary data and draws on the latest England and Wales Census [24] and the Index of Multiple Deprivation (IMD) for England 2010 [25]. Censuses have traditionally been a key source of localised information on the state of a nation's health [26]. Composite measures of socioeconomic deprivation are often used to capture the burden of socioeconomic adversity experienced by a neighborhood [27]. By drawing on a range of indicators, the IMD is considered to more precisely reflect the complex and multifactorial concept of area level deprivation. Area level deprivation indicators have been widely used in epidemiological research and have greatly facilitated research into the relationships between small area–level socioeconomic deprivation and poor health [23].

### 2.1. Limiting Long term Illness

The most recent census for England and Wales was collected in 2011 and included both a general health and a self-assessed limiting long-term illness (LLTI) question. A (LLTI) question has been included in the census since 1991 with data from this question historically being used by the Department for Health in their formula for funding local health services [26]. The information has also been used to allocate health resources within local jurisdictions and for policy development and monitoring, in relation to the assessment of progress towards better population health, the reduction of health inequalities, and improving access to services [24]. LLTI was chosen as the dependent variable as research has found it to have a positive relationship with area level deprivation [28], [29]. The wording of the 2011 Census question on LLTI was: “Are your day-today activities limited because of a health problem or disability which has lasted, or is expected to the last, at least 12 months? Include problems relating to old age?” with the possible answers being: 1. Yes, limited a lot, 2. Yes, limited a little, 3. No. In 2011, 8.3% of the population of England reported their daily activities were ‘limited a lot’ and this is the dependent variable of interest in this paper. With regard to the self-assessed aspect of the question, there is a wide body of research that supports the validity of self-assessed measures such as LLTI as indicators of health. Those who report an LLTI are more likely to die prematurely, to claim sickness benefits and to use medical services. Recent studies [30], [31] have shown that self-reported medical diagnoses can be a valid method of capturing health outcomes, in lieu of clinical scales, in the general population. In the UK, research by found that there are strong relationships between LLTI and other health outcomes including all cause and cause-specific mortality [32], [33].

Two further Census variables, the population aged 75 years plus and the percentage of residents reporting white ethnicity was also used to control for age and ethnic differences for each LSOA. Research has found increasing age is a significant predictor of LTTI outcomes. Including the percentage of individuals aged 75 plus, controls for the impact of age on LLTI outcomes across different areas. With regard to the inclusion of ethnicity as a predictor variable, research on health outcomes for different ethnicities in England has found that neighbourhood concentrations of people for ethnic minorities may be important for health [34]. Within this context, it is interesting to examine white ethnicity is a protector from LLTI at the same area level in Liverpool. With regard to spatial disaggregation, the Census for England and Wales contains a variety of spatial aggregates from the regional level down to the output area (OA). Super output areas (SOA) were designed to improve the reporting of small area statistics and are built up from groups of output areas (OA). The paper uses data at the lower super output area (LSOA). There are 32,844 LSOAs in England, with an average population of 1,500 individuals. There are 298 LSOAs in Liverpool. The LSOA level was chosen as the level of spatial analysis as this is the smallest level for which area level deprivation is available in England. From a statistical perspective, using rates of LLTI at the small area level as the dependent variable in an analysis may be problematic. Small populations naturally attract small numbers of events and this can pose problems in producing sufficient numbers for analysis, particularly where the phenomenon exhibits low incidence in the population [35]. This is particularly true for case specific events [35]. The LLTI question included in the Census of Population is a general question and specifically tells respondents to include LLTI problems relating to old age [26]. As such rates of responses to the LLTI question are much higher than for case specific diseases. Given the higher population numbers associated with LSOAs relative to postcodes or OAs, the issue of producing sufficient numbers for statistical analysis was therefore considered not to be a problem at the LSOA level.

### 2.2. Index of Multiple Deprivation (IMD)

The IMD is partially based on Census data and a combination of data derived from other sources such as the Inland Revenue, the Department of Health and the Department of Transport. The purpose of the IMD is to measure the multiple facets of deprivation at the small area level [27] and may be seen as a method to conceptualise ‘disadvantaged areas’ with respect to spatial concentrations of disadvantaged persons [27]. The IMD 2010 was constructed by combining seven general welfare domain scores weighted as followed; income (22.5%), employment (22.5%), housing and disability (13.5%), education, skills and training (13.5%), barriers to housing and services (9.3%), crime (9.3%), and living environment (9.3%). The IMD is based on data at the Lower Super Output Areas (LSOA) with each LSOA containing on average 1500 people. The IMD is available in two numerical forms; as a rank variable, which shows how an individual LSOA compares to other LSOAs in the country, and as an absolute score [27]. The LSOA ranked number one is the most deprived, with higher rankings indicating less deprived areas. The IMD score acts as the contextual, area level variable in this analysis.

### 2.3 Case Study Area: Liverpool

**Figure 1:**
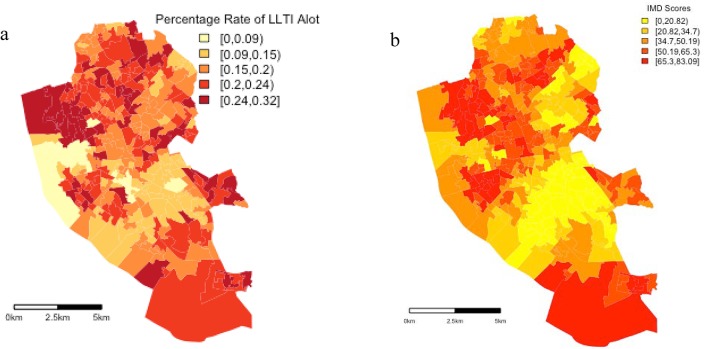
(a) Deprivation scores at the LSOA level in Liverpool and Figure 1 (b) Percentage of Individuals with a LLTI that ‘limits’ them a lot at the LSOA level in Liverpool

The principle aim of this study is to explore whether the impact of area level deprivation, often taken as a global parameter within regression frameworks, has a varying impact on LLTI within a specific city. In choosing a case study site, Liverpool has some of the highest rates of area level deprivation across England. At the same time, rates of LLTI are also higher than the national average across the city. Table 1 provides descriptive statistics for the dependent variable, the rate of LLTI at the LSOA level and independent variables; average IMD score, average percentage of individuals 75 years plus and average percentage of white ethnicity per LSOA for Liverpool. From Table 1 one can see that average deprivation (43) across the city is twice as high as the national average (21.6). More interesting to this paper is that on mapping the IMD scores at the LSOA level (Figure 1(a)), large variations in IMD scores can be observed across the city. Levels of deprivation vary from very high in South Liverpool and North Liverpool (65–83) to below the national average in central and East Liverpool. In comparison to a national LLTI rate of 8.3% across England, the average rate of LLTI at the LSOA level in Liverpool is 13%. Mapping LLTI at the LSOA level allows the spatial variability of LLTI to be examined across Liverpool. Figure 1(b) demonstrates that rates vary substantially across Liverpool. There is a cluster of LSOAs in the city centre with rates of LLTI less than 5%, while just north of the city centre rates are between 17% and 25%, three times the national average (Table 1). With regard to white ethnicity, Liverpool has a higher percentage of white ethnic residences, 89%, compared to the national average, 81. Regarding the percentage of the population 75 years of age plus, Liverpool actually has a lower percentage, 6.8% compared to the national average, 7.8%. Figure 1(a) and Figure 1(b) indicate that rates of both LLTI and deprivation vary across Liverpool.

**Table 1:** Summary Statistics for LLTI, IMD Score, Age and Ethnicity for Liverpool and England

	Liverpool Average	National Average
LLTI	12.7%	8.3%
IMD score	43	21.6
Percentage White Ethnicity	89%	81.5%
Percentage Aged 75 Plus	6.80%	7.8%

Examining Figure 1(a) and Figure 1(b), the spatial distribution of higher rates of LLTI maps onto higher rates of deprivation in South Liverpool. However, comparing rates of LLTI and deprivation for the North West Liverpool area, one can see that while the highest rates of LLTI are observed in this part of the city, deprivation rates in this area are only average to high relative to the South of Liverpool. This relationship is similarly observed in areas of North and North East Liverpool. This paper argues that the degree to which both these factors vary across space and there positive but nonlinear spatial relationship indicates Liverpool is a good case study to explore the use of a spatially explicit statistical framework to examine the relationship between area level deprivation and LLTI.

## 3. Methods: Geographically Weighted Regression

Ordinary least squares (OLS) regression has been commonly used in epidemiology to quantify relationships between exposures and health outcomes [13]. As noted in the Introduction, the underlying assumption of the global regression method is that the relationship under study is spatially constant. A global OLS regression can be specified as:

$$y_{i}=\beta_{o}+\sum_{k}\beta_{k}\chi_{ik}+\varepsilon_{i}$$ (1)

where*y*_*i*_*β*_*o*_ is the percentage of the population reporting a LLTI for each LSOA *i,* represents the intercept,*β*_*k*_ is the parameter estimate for variable *k*, and *χ*_*ik*_ is the value of the *k^th^* variable for *i*, and *ε*_*i*_ is the error term [36]. Thus, the estimated parameter from a global OLS model is spatially invariant. However, due to both the compositional and contextual profiles of differing areas, in reality the relationship between the dependent and independent variables should vary across space. OLS regression will therefore mask spatial variability in these relationships [9], [13]. Moreover, it ignores the spatial dependencies between variables (Huang and Leung, 2002), which may result in biased estimates and can result in overstated statistical significance of associations [13].

Geographically weighted regression (GWR) is a spatial statistics tool that expands standard regression for spatial data analysis by allowing for spatial variance in the estimated parameters [36]. GWR corrects for both spatial heterogeneity and spatial dependence of the data by estimating local, more robust parameters that capture the distinctiveness of place, and the spatial variations between dependent and independent variables [17]. This spatially explicit approach helps identify relationships that are likely masked by using traditional regression methods, that assume that the relationship between variables are the same throughout the entire study area [14],[20]. In contrast to the single regression observation calculated by an OLS model, GWR constructs a separate regression equation for each observation [20]. Each equation is calibrated using a different weighting of observations contained in the dataset [20]. The GWR model can be expressed as follows:

$$y_{i}=\beta_{o}(u_{i},v,)+\sum_{k}\beta_{k}(u_{i},v,)\chi_{ik}+\varepsilon_{i}$$ (2)

where (*u*_*i*_,*v*_*i*_) denotes the x and y coordinates of each LSOA centroid *i*. In GWR, the parameter estimates are made using an approach in which the contribution of a sample to the analysis is weighted based on its spatial proximity to the specific location under consideration. Thus the weighting of an observation is no longer constant in the calibration but may vary with different locations. Each LSOA centroid*, i,* is a regression point, and observations closer in proximity to *i* are weighted more than LSOA that are farther away [36]. Hence, neighboring LSOAs have more influence than distant LSOAs. An adaptive weighting scheme accounts for the variation in the size of LSOAs. The adaptive weighting function permits a larger bandwidth when the data are sparse and a small bandwidth when data are denser (36]. The bandwidth influences the weighting scheme, and is expressed in the same number of units included in the analysis. A large bandwidth approaches the parameter achieved in a global model, while a small bandwidth reveals local relationships [36]. The adaptive spatial kernel also ensures that an equal number of observations receive a non-zero weight value at each regression point [36]. The bi-square adaptive weighting scheme was used for the purpose of this paper. The Akaike Information Criterion (AICc) is used to determine the optimal kernel bandwidth size [36]. The model with the lowest AIC value indicates a better model fit [21], [36]. To test for spatial non-stationarity, a Monte Carlo significance test provides a randomized null hypothesis model, in which the location of each observation is arbitrarily assigned to the predictor and response variables. The null hypothesis assumes there is no significant difference in patterns of parameters estimates across place [18]. Hence, a significant Monte Carlo test reveals spatial variation in local response variables [36].

GWR is not without its limitations. The problem of collinearity amongst the predictor variables of a regression model has long been acknowledged and can lead to a loss of precision and power in the coefficient estimates [14]. This issue is heightened in GW regression since: (a) its effects can be more pronounced with the smaller samples that are used to calibrate each local regression; and (b) if the data is spatially heterogeneous in terms of its correlation structure, some localities may exhibit collinearity when others do not [38]–[40]. In both cases, (local) collinearity may cause serious problems in GW regression, when none are found in the corresponding global regression [38], [40]. Diagnostics to investigate local collinearity in a GW regression model, include finding: (i) local correlations amongst pairs of independent variables; (ii) local variance inflation factors (VIFs) for each independent variable; (iii) local variance decomposition proportions (VDPs) and (iv) local (design matrix) condition numbers (CNs) [40]. However, local correlations and local VIFs cannot detect collinearity with the intercept term [38], [40]. Thus, VDPs and CNs are considered superior diagnostics [40]. Reported VDFs greater than 10 indicate that multicollinearity between the independent variables is a problem [14], [40]

For the purpose of this paper, the GWmodel package in R software [40] is used to compute the results for the local regression models. The GWmodel package calculates the Akaike Information Criterion corrected (AICc) as a measure of goodness of fit for the global model and for the local GWR models. A second measure of goodness of fit compares the regression coefficients from the global and local models. If the quantity (2*standard error) for the global model parameter is less than the inter-quartile range for the equivalent local model parameter, the local model is probably a better fit. The VDF measure included in the GWmodel package is used to determine test for multicollinearity between the independent variables [40]. The packages RColorBrewer and classInt in the R software was used to develop each of the maps.

## 4. Results

As outlined in Section 3, OLS regression calculates one parameter estimate for each variable, and a single measure of model fit [14]. The global estimates for the entire study area are reported in Table 2. For every one-percentage increase in IMD score, the LSOA level LLTI rate increases by 0.17 %. This relationship is significant at the 0.01 level. The effect of ethnic composition, the percentage of white ethnic residents in the LSOA, on LSOA level adult LLTI is also significant, but at the 0.05 level. As the percentage of white ethnic residents increase, LLTI increases by 0.03%. For every one-percentage increase in the percentage of residents aged 75 years plus, the LSOA level LLTI rate increases by 0.79%. The age variable is also significant at the 0.01 level and has the strongest association with LLTI in the global model. The VIFs did not exceed the common threshold of 10; therefore there is no indication that multicollinearity is biasing the results [14]. Overall, the model is well specified and explains 78% of the variance in the LLTI rates at the LSOA level in Liverpool. This is very high R^2^ result, particularly relative to previous research that has compared estimates from OLS and GWR model [16], [22]. The AIC for the global regression model is 1,340.

**Table 2:** Global OLS Model of LLTI for Liverpool

	Coefficient	Standard error
Intercept	−3.233 ***	0.942
IMD score	0.174 ***	0.006
Percentage White Ethnicity	0.034 **	0.010
Percentage Aged 75 Plus	0.794 ***	0.045
R^2^	0.78	
AICc	1,340	
*** Significant at the 0.01 level, ** Significant at the 0.05 level		

Further analysis is needed to determine spatial heterogeneity in the outcome variable, and whether a local, nuanced model is more robust compared to the OLS model. The GWR 5-number summary and Monte Carlo significance tests for spatial heterogeneity of parameter test results are presented in Table 3. The Monte Carlo significance test, also available as part of the GWmodel package in R [40], indicates that all the parameters in the model are non-stationary across space. The AIC in the GWR model decreases to 1,119 and the adjusted r-square increases to 89% thus both indicators confirm that the GWR model is a better fit than the global model. The AIC determined bandwidth in the GWR model is 56 LSOAs. That is, each local GWR model is estimated on data from 56 LSOAs, which is less than 19% of the 298 LSOA in the global analysis.

**Table 3:** GWR Model of LLTI for Liverpool

	Min.	1^st^ Quartile	Median	3^rd^ Quartile	Max.	Test for Non-Stationarity (P-values)
Intercept	−22.79	−5.65	−0.83	3.99	31.53	0
IMD score	0.01	0.12	0.16	0.21	0.32	0
Percentage Aged 75 Plus	0.27	0.69	0.81	1.06	2.27	0
Percentage White Ethnicity	−0.31	−0.02	0.01	0.05	0.26	0
R^2^			0.89			
AICc			1,119			

**Figure 2:**
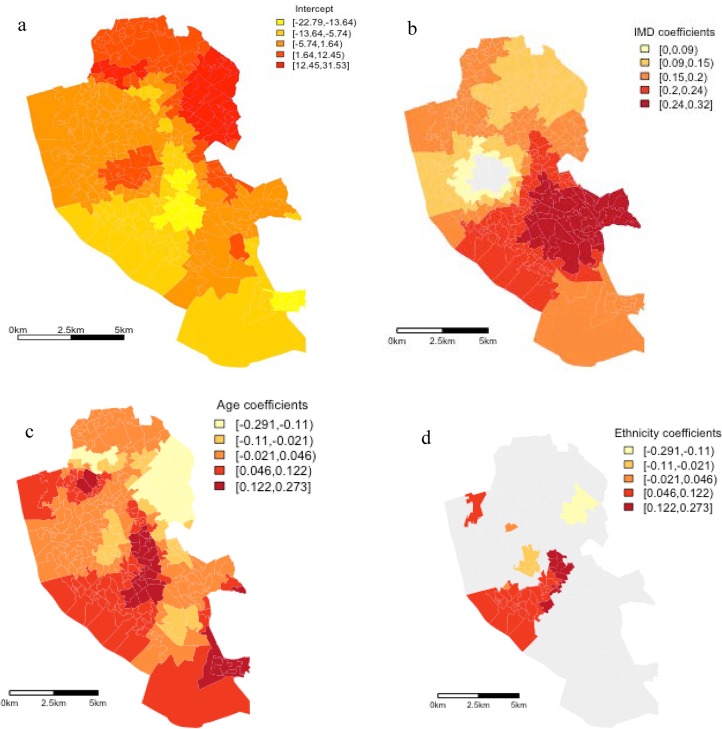
The spatial variation of the conditional parameters from GWR modeling

Table 3 shows that the effect of the covariates varies greatly across the study area. However, it is essential to map the local parameter estimated to observe where there is significant spatial heterogeneity between the independent and dependent variables. Figure 2 presents the variability for the intercept, and the significant (p < 0.05) coefficients for area level deprivation, the percentage of people aged 75 years plus and the percentage of the population of white ethnicity on LLTI at the LSOA level for Liverpool. Natural breaks were used to classify each category. From Figure 2(a) it can be observed that higher intercept values for LLTI are located in the North, particularly the North East of the city. This spatial trend implies that once spatial variations in the three explanatory variables in the model have been accounted for, rates of LLTI are higher in the North East of Liverpool. The relationship between LLTI and IMD score is shown in Figure 2(b). For area level deprivation only a small cluster of LSOAs has a non-significant effect on LLTI outcomes in Liverpool and similar to the OLS model all associations are positive. Figure 2 also indicates that most of central Liverpool reports significant coefficient greater than the global coefficient calculated by the OLS model. However, the magnitude of this association varies across the city. The highest deprivation coefficients (0.23–0.31) are observed in two areas, the East of Liverpool and around the city centre. This indicates that in these two areas higher IMD scores are related to increased LLTI rates compared to the South and North of the city. Continuing the analysis, Figure 2(c) presents the effects of the percentage population greater than 75 years old and LLTI. Similar to the deprivation coefficient there is significant geographic variation in the relationship between the increase in the percentage of the individual's aged 75 plus and LLTI across most of the study area. Controlling for deprivation and ethnicity, the highest effect is found in the North West of Liverpool. Again the effect of the age is greater in these areas than the average age coefficient (0.79) calculated by the global OLS model.

However, similar to deprivation the magnitude of these associations varies across Liverpool. The highest positive relationships are clustered in the North West of the city, indicating a higher percentage of residents aged 75 years and older are associated with higher levels of LLTI in this area. Residents aged 75 years and older in the South, Centre and North East of Liverpool, although also displaying a positive relationship, are associated with lower levels of LLTI. Finally, Figure 2(d) presents the results of the GWR model for deprivation and the percentage of white residents in the LSOA. Figure 2(d) indicates that there are fewer clusters of significant coefficients observed between the ethnicity proxy in an LSOA and LLTI, compared to the age and deprivation variables. However, unlike the age and deprivation variables, the ethnicity variable displays both positive and negative coefficient signs. In the North East of the city, a cluster of LSOAs are negatively associated with LLTI, that is a lower percentage of white residents in these LSOAs are associate with increasing rates of LLTI. In contrast, in a number of LSOAs in North Liverpool, and large parts of the city centre, a positive relationship is observed between ethnicity and LLTI. It is important to note that the global model only reported a small, positive relationship. Thus, if one only used a global OLS model the significant, negative relationship between ethnicity and LLTI in parts of Liverpool would go unobserved. Estimating the VDFs for each of the three explanatory variables using the GWmodel found that no LSOA reported a CN higher than 30. This indicates that multicollinearity is not a problem among the predictor variables.

## 5. Discussion

Over the last two decades, health geography has been successful in re-establishing interest in the role of place in shaping health and health inequalities [41]. However, this renewed interest has relied on conventional global regression models to isolate the “independent” contribution of place-level and individual-level factors [41], [42]. At the same time, these global regression techniques model space as a container, a discrete entity rather than a continuous parameter [41]. They do not account for the fact that place level factors can be interconnected [22]. GWR provides a technique for modelling space as a continuous parameter by incorporating a weighting function that gives the greatest weight to locations closest in space to the focal location, rather than resorting to arbitrarily defined administrative boundaries [22]. Mapping the intercepts from the GWR demonstrates that higher intercept values for LLTI are located in the North, particularly the North East of the city. This implies that once spatial variations in area level deprivation, age and ethnicity have been accounted for, rates of LLTI are higher in the North East of Liverpool. Mapping the explanatory variables, it was found that higher rates of area level deprivation and age profile were positively associated with LLTI across Liverpool. However, will the direction of the relationship for both variables was consistently positive across the city, the magnitude of the estimated coefficients varied across the city. Thus, while the global OLS model produced a high R^2^ (0.78), this research indicates that area level deprivation and the age profile of an area should not be modelled as global averages in Liverpool. The rationale for using spatially explicit modelling techniques is further confirmed on examining the coefficient for ethnicity. The estimated coefficient for ethnicity varies both in magnitude and direction; displaying a significant negative relationship with LLTI in one cluster of LSOAs in the North East of the city and a significant positive relationship in central Liverpool. Global OLS or MLM based estimates of area would have disguised this geographical variation. These findings point to the need to understand the heterogeneity of area level determinants of LLTI across geographical areas.

With regard to the magnitude of the deprivation coefficient it was found that the impact of area level deprivation is not linearly associated with the areas reporting the highest rates of deprivation. The South and North of Liverpool have some of England's highest area level deprivation rates. However, using GWR found that area level deprivation has a stronger relationship with LLTI rates in the East and the Centre of Liverpool compared to the more deprived South and North areas of the city. This is a very interesting result and requires consideration. We know that spatial differences in health reflect the socio-economic processes which systematically advantage or disadvantage different groups [43], [44]. Overall, Liverpool contains 6 of the 10 most deprived LSOAs in England. Rates of LLTI are also higher across Liverpool than the national average. Why then, is this relationship not linear at the local level in Liverpool? Why are relatively less deprived areas (although comparatively high in a national context) reporting a greater association between LLTI outcomes and area level deprivation?

One consideration is the data that is used in this analysis is problematic. Similar to the majority of research on neighbourhoods and health outcomes to date [41],[42],[44], this paper relies upon an index of deprivation that is based on available small area level data for the whole country, rather than an exhaustive list of social, economic, political and cultural factors that determine health outcomes within a specific area. Data limitations mean that important social factors linked to health outcomes such as social capital, kinship systems, shared histories, identity, etc are missing from these indexes [42]. Also missing from these datasets is information on how residents in an area or in a surrounding area perceive their own area [44]. Subjectively, do residents seem their area as deprived? The difference between objective and subjective perspectives of area level deprivation and health outcomes is important [44]. Research in London on mental health and adolescents London found that perceptions of area quality were linked to individual mental health, but that independently measured indicators of area deprivation were not so consistently associated with individual health outcomes [34]. Further research in the USA found independent associations between both objective and perceived neighbourhood quality and health [44], and highlight the particularly strong association between perceived neighbourhood quality and health. Although focused on active commuting rather than health outcomes, research in Paris [16] found that perception-based variables are also subject to non-stationarity. The first principle of ecological models is that multiple levels of factors, including individual and environmental ones, influence health behaviors [45]. Social relations or collective social functioning [42] within an area combined with missing information on how people perceive their neighbourhood may explain the attenuation of objective measures of deprivation in North West and South Liverpool. Thus, while GWR ensures that spatial dependency between variables is accounted for within this paper, we are still restricted by the data that is available to use.

Linking these results to public health policy, the study of relationships between place-level factors and health for public health policy is not new and evidence thereof is far from clear, and important issues, including the mechanism, measurement, and the analytical approach, require further exploration [43]. Furthermore, it is important to note that GWR is a method for hypothesis generating rather than hypothesis testing [43]. However, the spatial variability of the relative influence of individual and environmental factors presented here, suggest the importance of considering the local context in public health policy at the city level. Spatial heterogeneity in health outcomes is present within cities, as well as across cities. Within a public health context, this research indicates that even at the city level, a “one-size fits all” policy strategy is not the most appropriate approach to address health outcomes. Therefore city “wide” health polices need to be spatially adaptive, and relate to areas based on contextual similarities rather than artificial administrative boundaries. Small single patterns of non-stationarity remain difficult to interpret in detail [16]. However, the spatial clustering evident from mapping the results of the GWR indicates that models using traditional spatial boundaries such as LSOAs miss important contextual similarities between areas. GWR enables boundaries based on local contexts to be drawn, rather than relying on administrative boundaries. Incorporating these findings within a public health context may mean that the artificial boundaries relied on to date to model health outcomes should be dropped. Instead, GWR methods to identify common clusters of health associations should be used as a basis for identifying areas for local health interventions.
